# The global diffusion of organ transplantation: trends, drivers and policy implications

**DOI:** 10.2471/BLT.14.137653

**Published:** 2014-08-22

**Authors:** Sarah L White, Richard Hirth, Beatriz Mahíllo, Beatriz Domínguez-Gil, Francis L Delmonico, Luc Noel, Jeremy Chapman, Rafael Matesanz, Mar Carmona, Marina Alvarez, Jose R Núñez, Alan Leichtman

**Affiliations:** a Sydney Medical School, Charles Perkins Centre, The University of Sydney, New South Wales 2006, Australia.; b Department of Health Management and Policy, University of Michigan, Ann Arbor, United States of America (USA).; c Organización Nacional de Trasplantes, Madrid, Spain.; d Massachusetts General Hospital, Boston, USA.; e Service Delivery and Safety, World Health Organization, Geneva, Switzerland.; f Centre for Transplant and Renal Research, Westmead Hospital, Westmead, Australia.; g Department of Internal Medicine, University of Michigan, Ann Arbor, USA.

## Abstract

Rising incomes, the spread of personal insurance, lifestyle factors adding to the burden of illness, ageing populations, globalization and skills transfer within the medical community have increased worldwide demand for organ transplantation. The Global Observatory on Donation and Transplantation, which was built in response to World Health Assembly resolution WHA57.18, has conducted ongoing documentation of global transplantation activities since 2007. In this paper, we use the Global Observatory’s data to describe the current distribution of – and trends in – transplantation activities and to evaluate the role of health systems factors and macroeconomics in the diffusion of transplantation technology. We then consider the implications of our results for health policies relating to organ donation and transplantation. Of the World Health Organization’s Member States, most now engage in organ transplantation and more than a third performed deceased donor transplantation in 2011. In general, the Member States that engage in organ transplantation have greater access to physician services and greater total health spending per capita than the Member States where organ transplantation is not performed. The provision of deceased donor transplantation was closely associated with high levels of gross national income per capita. There are several ways in which governments can support the ethical development of organ donation and transplantation programmes. Specifically, they can ensure that appropriate legislation, regulation and oversight are in place, and monitor donation and transplantation activities, practices and outcomes. Moreover, they can allocate resources towards the training of specialist physicians, surgeons and transplant coordinators, and implement a professional donor-procurement network.

## Résumé

La hausse des revenus, le développement des assurances personnelles, les facteurs de mode de vie ajoutant à la charge de morbidité des maladies, le vieillissement des populations, la mondialisation et le transfert des compétences au sein de la communauté médicale ont augmenté la demande mondiale de transplantation d'organe. L'Observatoire Mondial du Don et de la Transplantation, qui a été fondé en réponse à la résolution WHA57.18 de l'Organisation mondiale de la Santé, a rassemblé une documentation sur les activités de transplantation dans le monde de façon continue depuis 2007. Dans cet article, nous utilisons les données de l'Observatoire Mondial pour décrire la distribution actuelle (et les tendances) des activités de transplantation et pour évaluer le rôle des facteurs de systèmes de santé et de la macroéconomie dans la diffusion des technologies de transplantation. Nous considérons ensuite les implications de nos résultats sur les politiques de santé relatives au don et à la transplantation d'organe. La majorité des États Membres de l'Organisation mondiale de la Santé s'engagent maintenant dans la transplantation d'organe et plus d'un tiers d'entre eux ont réalisé des transplantations avec des organes provenant de donneurs décédés en 2011. En général, les États Membres qui se sont engagés dans la transplantation d'organe, ont un meilleur accès aux services médicaux et des dépenses totales de santé plus élevées par habitant que les États Membres où la transplantation d'organe n'est pas réalisée. La disponibilité de la transplantation avec des organes provenant de donneurs décédés était étroitement associée avec des niveaux élevés de revenu national brut par habitant. Il existe plusieurs manières possibles pour les gouvernements de soutenir le développement éthique des programmes de don et de transplantation d'organe. En particulier, ils peuvent s'assurer que la législation, la réglementation et la surveillance sont en place, et contrôler les activités, les pratiques et les résultats des dons et des transplantations. En outre, ils peuvent affecter des ressources pour la formation des médecins spécialistes, des chirurgiens et des coordinateurs de transplantation, et mettre en œuvre un réseau professionnel de recrutement des donneurs.

## Resumen

El aumento de la renta, la proliferación de los seguros personales y los factores del estilo de vida, sumados a la carga de enfermedades, el envejecimiento de la población, la globalización y la transferencia de conocimientos en la comunidad médica, han aumentado la demanda mundial de trasplantes de órganos. El Observatorio Mundial de Donación y Trasplante, creado en respuesta a la resolución WHA57.18 de la Asamblea Mundial de la Salud, ha llevado a cabo una documentación continua de las actividades mundiales de trasplantes desde 2007. En este informe, se emplean los datos del Observatorio Global para describir la distribución actual (y las tendencias) de las actividades de trasplante y para evaluar el papel de los factores de los sistemas sanitarios y de la macroeconomía en la difusión de la tecnología de trasplante. A continuación, se consideraron las repercusiones de los resultados en las políticas de salud relacionadas con la donación y el trasplante de órganos. En la actualidad, la mayoría de los Estados miembros de la Organización Mundial de la Salud participa en el trasplante de órganos y más de un tercio realizó trasplantes de donantes fallecidos en 2011. En general, los Estados miembros que participan en el trasplante de órganos cuentan con mayor acceso a los servicios médicos y tienen un mayor gasto total en salud per cápita que los Estados miembros donde no se realizan el trasplantes de órganos. La prestación de los trasplantes de donantes fallecidos se asoció estrechamente con altos niveles de renta nacional bruta per cápita. Existen varias formas en que los gobiernos pueden fomentar el desarrollo ético de los programas de donación y trasplante de órganos. En concreto, pueden garantizar que se adopte una legislación, regulación y supervisión adecuadas, así como realizar un seguimiento de las actividades, las prácticas y los resultados de la donación y el trasplante. Además, pueden destinar recursos a la formación de médicos especialistas, cirujanos y coordinadores de trasplantes, así como poner en marcha una red profesional de adquisición de donantes.

## ملخص

أدت زيادة الدخول وانتشار التأمين الشخصي والعوامل المؤثرة في أنماط العيش التي تضيف إلى عبء الاعتلال وزيادة أعمار السكان والعولمة ونقل المهارات داخل المجتمع الطبي إلى ازدياد الطلب العالمي على زرع الأعضاء. ويجري المرصد العالمي للتبرع بالأعضاء وزرعها، الذي تم تأسيسه استجابة لقرار جمعية الصحة العالمية، ج ص ع **57-18، ** توثيقًا لأنشطة زرع الأعضاء على الصعيد العالمي منذ عام **2007 **. ونستخدم في هذه الورقة بيانات المرصد العالمي لوصف التوزيع الراهن لأنشطة زرع الأعضاء والاتجاهات في هذه الأنشطة وتقييم دور عوامل النظم الصحية والاقتصاد الكلي في انتشار تكنولوجيا زرع الأعضاء. ونقوم بعد ذلك بدراسة آثار نتائج السياسات الصحية ذات الصلة بالتبرع بالأعضاء وزرعها. وتشارك معظم الدول الأعضاء في منظمة الصحة العالمية في الوقت الراهن في زرع الأعضاء، وأجرى ما يزيد عن ثلث هذه الدول زرع أعضاء من متبرعين متوفين في عام **2011 **. وبشكل عام، يزداد وصول الدول الأعضاء التي تشارك في زرع الأعضاء إلى خدمات الأطباء ويزداد الإنفاق الصحي الإجمالي للفرد لدى هذه الدول عنه لدى الدول الأعضاء التي لا يجرى بها زرع الأعضاء. وارتبط توفير زرع الأعضاء من متبرعين متوفين على نحو وثيق بارتفاع مستويات الناتج القومي الإجمالي للفرد. وتوجد طرق عديدة يمكن للحكومات من خلالها دعم التطور الأخلاقي لبرامج التبرع بالأعضاء وزرعها. على وجه التحديد، يمكنها ضمان تطبيق التشريعات واللوائح التنظيمية والمراقبة ورصد الأنشطة والممارسات والحصائل فيما يخص التبرع بالأعضاء وزرعها. علاوة على ذلك، يمكنها تخصيص الموارد لتدريب الأطباء الأخصائيين والجراحين ومنسقي زرع الأعضاء وإنشاء شبكة مهنية لتأمين الحصول على المتبرعين.

## 摘要

收入的增加、个人保险的普及、疾病负担中增加的生活方式因素、人口老龄化、全球化以及医疗社区内的技术转移都在增加全球对器官移植的需求。响应世界卫生大会WHA57.18决议而建立的全球捐献和移植瞭望台自2007年以来就一直对全球移植活动进行文档编制。在本文中，我们使用全球瞭望台的数据来描述移植活动当前的分布和趋势，并评价卫生系统因素和宏观经济学在移植技术普及中的作用。而后，我们思考与器官捐献和移植相关的卫生政策的结果对我们的影响。现在世界卫生组织的成员国大多数都有从事器官移植，在2011年超过三分之一成员国实施已故供体移植。一般来说，较之不实施器官移植的成员国，实施器官移植的成员国拥有更多的医生服务和人均医疗支出总额。已故供体移植提供与高水平人均国民总收入密切相关。政府有几种方法来支持器官捐献和移植项目的伦理发展。具体来说，他们可以确保适当的立法、监管和监督，并监控捐献和移植活动、实践和结果。此外，他们可以使资源分配向培训专家医生、外科医生和移植协调人员倾斜，并实施专业的供体获取网络。

## Резюме

Рост доходов, распространение индивидуального страхования, факторы образа жизни, влияющие на уровень заболеваемости, старение населения, глобализация и передача навыков среди медицинского сообщества повысили всемирную потребность в трансплантации органов. Глобальная обсерватория по донорству и трансплантации, которая была создана во исполнение резолюции WHA57.18 Всемирной ассамблеи здравоохранения, осуществляет постоянное ведение документации по трансплантации во всем мире с 2007 года. В данной работе использованы данные Глобальной обсерватории для описания распространения и тенденций в сфере трансплантологии и для оценки влияния факторов в системе здравоохранения и макроэкономике на распространение технологий трансплантации. Также рассматривается влияние полученных результатов на развитие политик здравоохранения в отношении донорства и трансплантации. Большинство стран, являющихся членами Всемирной организации здравоохранения, занимаются трансплантацией органов, более трети этих стран осуществляли трансплантацию органов от мертвых доноров в 2011 году. В общей сложности, страны-члены ВОЗ, осуществляющие трансплантацию органов, имеют более высокий уровень доступа к медицинским услугам и более высокий уровень расходов на здравоохранение на душу населения, чем страны-члены ВОЗ, где трансплантация органов не производится. Осуществление трансплантации органов от мертвых доноров тесно связано с высоким уровнем национального валового дохода на душу населения. Существует несколько способов, с помощью которых правительство может поддержать этичное развитие программ донорства и трансплантации органов. А именно, правительство может обеспечить наличие необходимого законодательства, нормативных положений и методов надзора, а также осуществлять контроль деятельности по донорству и трансплантации органов, практических методов и результатов. Более того, оно может предоставлять ресурсы для обучения квалифицированных терапевтов, хирургов и трансплантационных координаторов и обеспечить функционирование профессиональных донорских сетей.

## Introduction

In May 2004 the World Health Assembly adopted resolution WHA57.18, in recognition of the global increase in transplantation activities, the associated risks to patient safety, the trafficking of organs for transplantation and the trafficking of human beings as sources of such organs. This resolution urged the World Health Organization’s (WHO’s) Member States to implement “effective national oversight of the procurement, processing and transplantation of human cells, tissues and organs” and requested the collection of global data on practices in allogeneic transplantation and their outcomes.^1^ In response, the Global Observatory on Donation and Transplantation was established as an official collaboration between WHO and the *Organización Nacional de Trasplantes*.^2^ In 2011, the Global Observatory contained information on allogenic donation and transplantation activities for 105 Member States, including records of 112 939 solid organ transplants performed in 2011.^3^

In this article, we used Global Observatory data to investigate the current distribution of global transplantation activities and the temporal trends in rates of solid organ transplantation for each Global Burden of Disease region.^4^ We identified the Member States driving these trends and the health policies that were associated with substantial increases in transplantation activities between 2006 – i.e. the first year for which the Global Observatory collected comprehensive data – and 2011. We also evaluated the broad macroeconomic and health system determinants of the diffusion of the practice of organ transplantation.

International variation in transplantation activities is recognized to be largely unrelated to the actual distribution of medical need – correlating instead with the resources available for health-care provision.^5^^,^^6^ In previous studies of countries with established programmes of renal replacement therapy, the incidence of dialysis and kidney transplantation in a given country has been found to be significantly associated with that country’s gross domestic product (GDP) per capita and the percentage of the GDP spent on health care – but not with demographic characteristics or the underlying risk factor burden.^5^ These observations are perhaps unsurprising since, in general, higher income per capita and higher levels of health spending are associated with greater access to expensive, resource-intensive medical technologies, such as transplantation.^7^ However, there are indications that the level of correlation between income per capita and transplantation activity has diminished over the last few decades. For example, in a study of the diffusion of kidney transplantation – from 1975 to 1995 – across the countries belonging to the Organisation for Economic Co-operation and Development, significant convergence was observed in the number of transplants performed per country but not in GDP per capita.^8^ In the present study, therefore, we examined whether income per capita remains a determinant of the existence and capacity of transplant programmes across the WHO’s Member States. We also investigated the relationships between transplantation activity and health system factors including the number of physicians per capita, total health expenditure, public health expenditure and out-of-pocket payments.

Previous studies on this topic have focused on the number of kidney transplants per million population per year as the outcome.^5^^,^^8^ However, this approach excludes all countries that do not currently engage in kidney transplantation and is not ideal for describing countries that have only recently begun to practise transplantation. We therefore used an alternative method for evaluating the global diffusion of transplantation technology. This method was based on categorical levels of health system capacity with respect to solid organ transplantation (Box 1). The designation of levels of health system capacity – as a framework by which to evaluate the stage of development of national organ donation and transplantation programmes – was proposed during the WHO Madrid Consultation in 2010.^9^ By applying the Global Observatory data to such a framework, we broadly describe where each Member State stands with respect to the goal of transplantation self-sufficiency – i.e. the provision of a sufficient number of organs for residents in need, from within the country or through regional cooperation.^10^

Box 1 **Definitions^a^ of hierarchical levels of capacity with respect to the provision of organ donation and transplantation services in a given country**

**Level 1**

No local transplantation activity – either reported to the Global Observatory on Donation and Transplantation between 2006 and 2011 or detected by additional investigation.

**Level 2**

At least one kidney transplant centre – with the capacity to perform living donor nephrectomy, kidney transplantation and post-transplant management of recipients – within the country’s borders. No deceased donor activity reported to the Global Observatory on Donation and Transplantation between 2006 and 2011.

**Level 3**

Countries that have commenced deceased donor kidney transplantation within their own borders. Sufficient local capacity – including local medical expertise – exists to perform kidney recovery surgery from deceased and living donors, kidney transplantation and recipient management. Activities may also include liver transplantation and isolated cases of heart and lung transplantation.

**Level 4**

Deceased donor kidney and liver transplantation have been performed for at least five years. Heart and lung transplantation also available, either locally or via formal international cooperative organ-sharing agreements such as Eurotransplant and Scandiatransplant. Legislation permits and regulates organ donation and transplantation.

**Level 5**

An established multi-organ deceased donor transplant programme exists that is capable of providing kidney, liver, heart, lung and pancreas transplantation either locally or via formal international cooperative organ-sharing agreements. The transplant programme has been providing multi-organ deceased donor transplants consistently for at least five years, with an overall rate of transplantation in 2010 above 30 solid organ transplants per million population. The country has a government-recognized authority that is responsible for oversight of organ donation and transplantation activities.

^a^ Levels of health system capacity proposed during the WHO Madrid Consultation in 2010.^9^

## Trends in transplantation activities

Counts for living and deceased donor kidney, liver, pancreas, heart, lung and small bowel transplants performed between 2006 and 2011 were obtained from the Global Observatory database.^3^ Each year, for each of the WHO’s 194 Member States, the Global Observatory sends a standardized questionnaire to a relevant national focal point or a person officially designated by the relevant Ministry of Health.^11^ Activity data were available for 105 Member States in 2011, including five – Bhutan, Cameroon, Ethiopia, Fiji and the Maldives – that reported no transplantation activity. Forward interpolation was used from year to year to minimize missing data. The 10 Member States reporting the highest absolute numbers of living donor transplants in 2011 were the United States of America (*n* = 6020), India (*n* = 5482), Turkey (*n* = 3044), Mexico (*n* = 1894), Egypt (*n* = 1867), Japan (*n* = 1850), Brazil (*n* = 1748), Republic of Korea (*n* = 1620), Islamic Republic of Iran (*n* = 1545) and the United Kingdom of Great Britain and Northern Ireland (*n* = 1063). The 10 Member States with the highest deceased donor transplant numbers were the United States (*n* = 23 368), China (*n* = 6806), Brazil (*n* = 5097), France (*n* = 4634), Germany (*n* = 4064), Spain (*n* = 3886), the United Kingdom (*n* = 3048), Italy (*n* = 3020), Canada (*n* = 1738) and Poland (*n* = 1446).

**Fig. 1:**
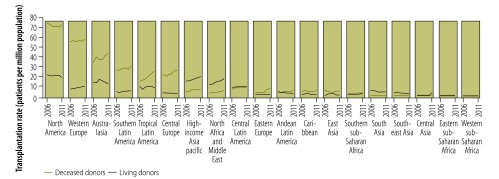
Distribution of solid organ transplantation activity, by region used in the Global Burden of Disease Study, 2006–2011

Notes: Data on Member States were grouped according to the regions used in the Global Burden of Disease Study (https://www.healthdata.org/sites/default/files/files/GBD_GBD2010_Regions_countries.pdf).

Fig. 1 shows the distribution of solid organ transplantation activities across regions specified by the Global Burden of Disease study. Both living and deceased donor transplantation activity increased in north Africa and the Middle East between 2006 and 2011 (Fig. 1). These regional increases were driven predominantly – in the case of deceased donor transplantation – by activities in the Islamic Republic of Iran and Turkey, and – in the case of living donor transplantation – by activities in Jordan and Saudi Arabia.^13^ Turkey experienced large increases in transplantation activity following the establishment of its National Coordination Centre in 2001. The establishment of this centre brought Turkish organ procurement and transplantation under the control of the national Ministry of Health and reoriented donation and transplantation around hospital-based transplant coordinators.^14^ Similar reforms to systems for donor identification, management and organ recovery in the Republic of Korea^15^ were probably important contributors to the increases in transplantation activity also observed for the high-income Asia Pacific region between 2006 and 2011.

Oceania and central sub-Saharan Africa are not shown as no transplantation activity was recorded for either of these regions between 2006 and 2011. National counts for all living and deceased donor transplants – kidney, liver, heart, pancreas, lung and small bowel – were obtained from the Global Observatory on Donation and Transplantation database.^3^

Activity data were available for 105 Member States in 2011. If activity data were missing for a given Member State in a given year, then the number of transplants performed in the previous year was carried over. Member States that did not report to the Global Observatory on any occasion between 2006 and 2011 were assumed to have no transplantation activity over that period.

Estimates of population size for each year between 2006 and 2011 were taken from the United Nations World Population Prospects, 2012 Revision,^12^ and regional transplantation rates were calculated by taking the total regional population, in millions, as the denominator.

In Australasia, the rate of deceased donor transplantation increased after 2008, coinciding with the establishment of an official authority responsible for the national coordination of donation and transplantation systems.^16^ In tropical Latin America and central Europe, increasing rates of deceased donor transplantation were driven predominantly by the trends in Brazil and Croatia, respectively. The rate of deceased donor transplantation in Brazil increased after 2005, when the Ministry of Health established that all hospitals with more than 80 beds should have an internal donation and transplantation commission.^17^ In Croatia, the rate of deceased donor transplantation increased more than 10-fold in the decade ending in 2011 as the result of several reforms – including the appointment of hospital and national transplant coordinators, the introduction of reimbursement for donor hospitals, public awareness campaigns, participation in cross-border organ sharing through Eurotransplant, and updated legislation.^18^

The centralization of the coordination of organ donation and transplantation under an officially recognized authority, the reorientation of organ recovery around transplant coordinators and the systematization of donor identification and organ recovery are all key components of the frequently cited “Spanish Model” of organ donation and transplantation.^19^^,^^20^ The successful implementation of these policies by a diverse range of countries – and the impact on rates of deceased donor transplantation between 2006 and 2011 – are evidence of the potential effectiveness and reproducibility of the Spanish Model.

Declining rates of living donor transplantation were observed in south Asia and south-east Asia, where these trends were largely driven by reduced activity in Pakistan and the Philippines, respectively. The declining rate of deceased donor transplantation observed in east Asia was driven by reduced activity in China. Pakistan signed into law the Ordinance on Human Cell and Tissue Transplantation in 2010, thus criminalizing organ sales.^21^ The Philippines implemented an expanded anti-human trafficking law in 2009.^21^ Parallel efforts to curb transplant tourism by major exporters of recipients have also influenced these trends.^22^ In China, declining rates of deceased donor transplantation coincided with a shift away from donation by executed prisoners, the implementation of laws limiting transplant tourism, and the closure of transplant programmes that failed to comply with new regulations.^23^ China is now in the process of implementing a new national programme of deceased donor transplantation that is based on a network of hospital-based organ procurement organizations, with oversight from national committees accountable to the Ministry of Health.^24^

## Global diffusion

As at 31 December, 2011, the Global Observatory had recorded activity of at least one organ transplant in 100 Member States, including deceased donor transplantation activity in 69 Member States in the year 2011. Another 11 Member States – Bahrain, Bosnia and Herzegovina, Honduras, Iraq, Jamaica, Kazakhstan, Montenegro, Serbia, Trinidad and Tobago, Ukraine and Viet Nam – were identified, via expert review or literature and web-based searches, as currently being engaged in transplantation activity. Therefore, most (57%) of the WHO’s Member States were engaged in some level of organ transplantation activity between 2006 and 2011, and over a third (36%) reported deceased donor transplantation activity in 2011.

Major geographical disparities in access to transplantation persist: 62% of the 112 939 solid organ transplants reported in 2011 were performed in high-income Member States, while only 28%, 9% and less than 1% were performed in upper-middle-, lower-middle- and low-income Member States, respectively. It is, however, noteworthy that, although the majority of organ transplantation takes place in high-income Member States, the practice of organ transplantation has now diffused across all income strata and has reached the populations of low-income Member States including Bangladesh, Kenya, Kyrgyzstan, Myanmar, Nepal and Tajikistan.

## Macroeconomic and health system factors

**Fig. 2:**
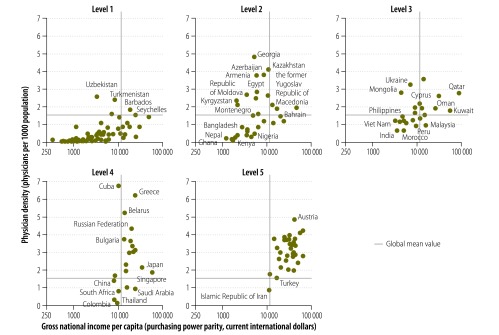
Gross national income per capita, physician density and capacity for solid organ transplantation, Member States of the World Health Organization, 2006–2011

Notes: The appropriate designation for each Member State to the five levels of transplantation capacity indicated was largely determined based on transplantation activities reported to the Global Observatory on Donation and Transplantation (Box 1). However, extra ascertainment – expert review and literature and web-based searches – was used to identify any additional Member States that engaged in organ transplantation between 2006 and 2011. Further details of country classifications are available from the corresponding author.

We divided Member States into five levels depending on their transplantation capacity, with levels 1 and 5 representing Member States with the lowest and highest transplantation capacities, respectively (Box 1). Fig. 2 shows, for each level of transplantation capacity, the correlation between gross national income per capita – measured, in terms of purchasing power parity, in international dollars – and physician-to-population ratio. For the majority of the 76 Member States not reporting any transplantation activity – i.e. those assigned to level 1 – gross national income per capita and physician-to-population ratio were generally below the global mean values, of 12 000 International dollars and 1.5 physicians per 1000 population, respectively. Level 2 Member States (*n* = 34), defined as having one or more centres providing living kidney transplantation, tended to have higher per capita income and notably higher physician-to-patient ratios compared with level 1 Member States. Exceptions included Bangladesh, Ghana, Kenya, Nepal, Nigeria, Pakistan and Sudan. The 23 Member States that were assigned to level 3, based on deceased donor transplantation activity, tended to have higher per capita incomes than level 2 Member States. Most level 4 Member States (*n* = 21) had per capita incomes and physician-to-population ratios above the global means – the exceptions being China, Colombia, South Africa and Thailand. Thirty-two of the 33 Member States assigned to level 5 had two or more physicians per thousand population and gross national incomes that exceeded 12 000 international dollars per capita – the only exception was the Islamic Republic of Iran. In logistic regression analyses of these data, higher physician-to-population ratio – but not higher gross national income per capita – was found to be significantly associated with the existence of any transplantation activity (Table 1). Among the Member States with any transplantation activity, however, the existence of deceased donor transplantation activity was found to be significantly associated with higher gross national income per capita, but not with higher physician-to-population ratio (Table 1).

Data on gross national income per capita – measured as purchasing-power parity, in international dollars – and the number of physicians per 1000 population were obtained from the World Bank.^25^ Forward interpolation was used to minimize missing data. All values are from 2011 or the most recent prior year for which data were available. If no data were available between 2000 and 2011 for a given variable, then the value was treated as missing for that country (*n* = 16). Andorra, Cook Islands, Democratic People's Republic of Korea, Monaco, Niue, San Marino and South Sudan were excluded because of small population size, data unavailability, or both.

**Table 1:** Association between solid organ transplantation activity and macroeconomic and health-system factors, 2006–2011

Predictor	OR (95% CI)	
	Any transplantation activity (*n* = 187)^a^	Deceased donor transplantation activity (*n* = 111)^a,b^
**Macroeconomics versus health services**^c^		
GNI per capita, in international dollars (per unit increase in the natural log)	1.31 (0.70–2.46)	3.16 (0.97–10.28)
Physicians per 1000 population (per additional physician per 1000)	10.1 (2.01–50.3)	0.61 (0.17–2.20)
**Total versus out-of-pocket health expenditure^d^**		
Total health expenditure (per unit increase in the natural log)	3.60 (1.82–7.12)	3.79 (0.98–14.65)
Out-of pocket expenditure as a percentage of total health expenditure (per 10% increase)	1.17 (0.42–3.20)	1.07 (0.14–8.30)
**Total versus public health expenditure**^e^		
Total health expenditure (per unit increase in the natural log)	4.86 (1.48–15.89)	3.81 (0.59–24.69)
Total health expenditure, per cent public (per 10% increase)	0.73 (0.27–2.00)	1.08 (0.14–8.06)

CI: confidence interval; GNI: gross national income; OR: odds ratio.

^a^ The models were run for the World Health Organization’s Member States. Andorra, Cook Islands, Democratic People's Republic of Korea, Monaco, Niue, San Marino and South Sudan were excluded because of small population or the incompleteness of the available data or both.

^b^ Conditional on any transplantation activity.

^c^ ORs adjusted for both variables and for the interaction between GNI and physicians per 1000 population.

^d^ ORs adjusted for both variables and for the interaction between total and out-of-pocket health expenditures.

^e^ ORs adjusted for both variables and for the interaction between total health expenditure and the percentage of total health expenditure accounted for by public funds.

**Fig. 3:**
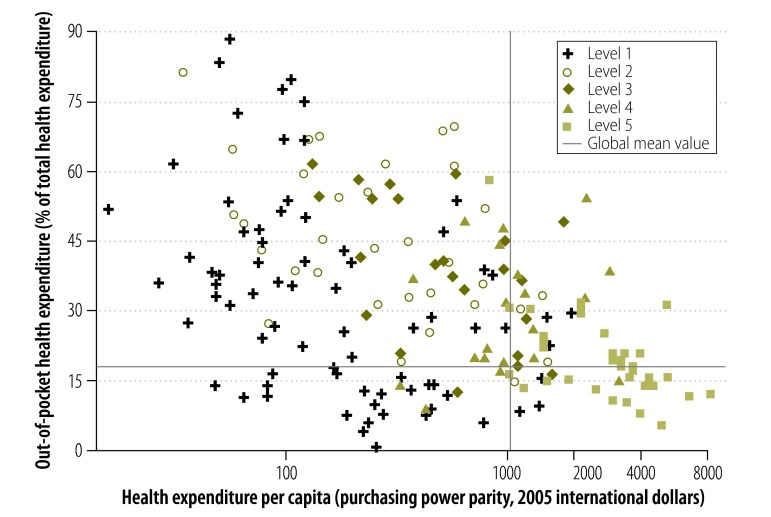
Total and out-of-pocket health expenditure and capacity for solid organ transplantation, Member States of the World Health Organization, 2011

Notes: The appropriate designation for each Member State to the five levels of transplantation capacity indicated was largely determined based on transplantation activities reported to the Global Observatory on Donation and Transplantation (Box 1). However, extra ascertainment – expert reviews and literature and web-based searches – was used to identify any additional Member States that engaged in organ transplantation between 2006 and 2011. Further details of country classifications are available from the corresponding author.

Fig. 3 shows the relationships between out-of-pocket expenditure on health, total health expenditure per capita and level of transplantation capacity. Member States with the highest level of transplantation capacity – i.e. those assigned to level 5 – tended to have relatively high total health expenditures and relatively low out-of-pocket expenditures. Member States that had no transplantation activity, or living donor transplantation activity only, tended to have below-average health expenditures – but showed no clear trend with respect to out-of-pocket expenditures. Logistic regression confirmed that there was no significant association between the existence of transplantation activity in a Member State and the magnitude of out-of-pocket expenditures (Table 1). In contrast, after adjusting for out-of-pocket expenditure, higher total health expenditure per capita was associated with a significant increase in the likelihood of any transplantation activity and with a nonsignificant increase in the likelihood of having initiated deceased donor transplantation (Table 1).

Economic data were obtained from the World Bank.^25^ Forward interpolation was used to minimize missing data. All values are from 2011 or the most recent prior year for which data were available. If no data were available between 2000 and 2011 for a given variable, then the value was treated as missing for that country (*n* = 16). Andorra, Cook Islands, Democratic People's Republic of Korea, Monaco, Niue, San Marino and South Sudan were excluded because of small population size, data unavailability, or both.

**Fig. 4:**
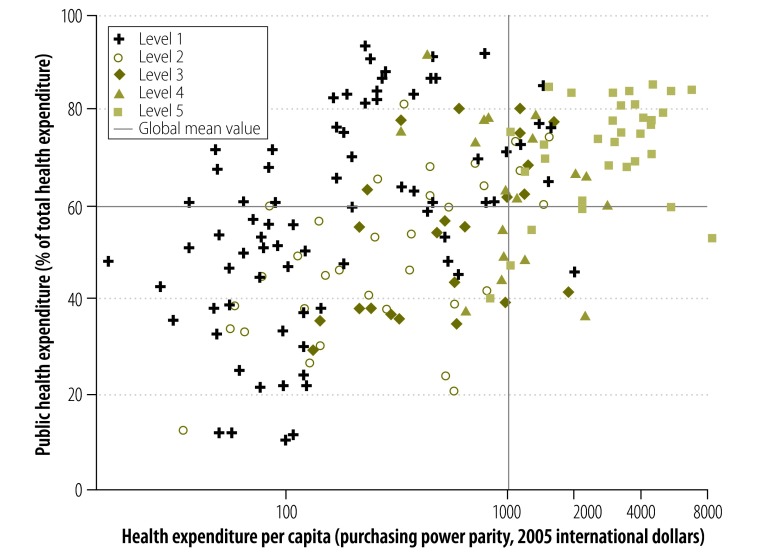
Total health expenditure, public health expenditure as a proportion of total health expenditure and capacity for solid organ transplantation, Member States of the World Health Organization, 2011

Notes: The appropriate designation for each Member State to the five levels of transplantation capacity indicated was largely determined based on transplantation activities reported to the Global Observatory on Donation and Transplantation (Box 1). However, extra ascertainment – expert reviews and literature and web-based searches – was used to identify any additional Member States that engaged in organ transplantation between 2006 and 2011. Further details of country classifications are available from the corresponding author.

Fig. 4 shows the relationships between the proportions of total health expenditure accounted for by public funds, total health expenditure per capita and level of transplantation capacity. Member States with the highest level of transplantation capacity tended to have relatively high proportions of their health expenditures accounted for by public funds and relatively high health expenditures per capita. However, there was no evidence of an association between the existence of transplantation activity in a Member State and public health expenditure as a percentage of total expenditure. This observation is consistent with previous findings that showed that total health expenditure – but not the public share of health-care expenditure – was independently associated with international variation in rates of treatment for end-stage kidney disease.^5^ These findings probably reflect diversity in the extent to which the private sector participates in the delivery of renal replacement therapy.

Economic data were obtained from the World Bank.^25^ Forward interpolation was used to minimize missing data. All values are from 2011 or the most recent prior year for which data were available. If no data were available between 2000 and 2011 for a given variable, then the value was treated as missing for that country (*n* = 16). Andorra, Cook Islands, Democratic People's Republic of Korea, Monaco, Niue, San Marino and South Sudan were excluded because of small population size, data unavailability, or both.

Overall, our observations indicate that, in general, transplanting Member States have relatively high health expenditures per capita and populations with relatively good access to physician services – two factors that are likely to indicate a minimum standard of available tertiary care. Notable outliers to this observation included the former Soviet countries of central Asia and eastern Europe, where physician to population ratios are high yet transplantation capacities are relatively low. Low physician wages, informal payments and negative public attitudes towards organ donation and transplantation potentially contribute to this observation.^26^^,^^27^ The situation in this region has begun to improve, however. The north-west region of the Russian Federation recently introduced a transplant coordination model that is having a positive impact on the region’s organ donation and transplantation trends.^26^ Armenia, Belarus, Georgia, Kazakhstan, the Republic of Moldova and Tajikistan have also taken steps towards modernizing their organ procurement and transplant systems.^28^

**Fig. 5:**
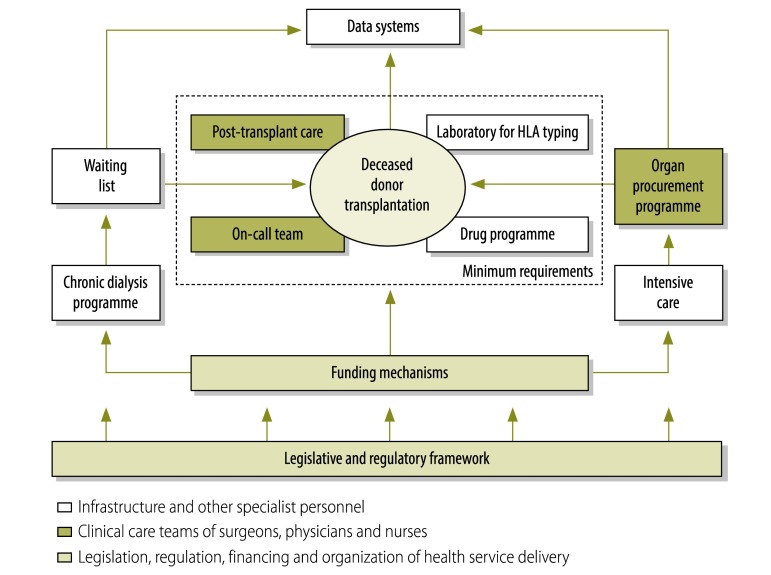
Schematic of the minimum health system requirements for performing deceased donor organ transplantation

We also observed that, among transplanting countries, provision of deceased donor transplantation remains significantly associated with gross national income per capita. This reflects the extra resources and organization needed to support deceased donor transplantation, including the requirements for a waiting list and allocation system, an organ procurement programme, an on-call transplantation team and relevant intensive-care resources (Fig. 5).^29^ For many low- and middle-income countries, the costs of post-transplantation care and ongoing immunosuppression present a substantial additional barrier to the development of greater transplantation capacity.

HLA: human leukocyte antigen.

Lastly, it is worth reflecting on the observation that a substantial proportion of global transplantation activity takes place in countries where out-of-pocket expenditure on health-care services exceed the global mean. In this context, the initiation and development of organ transplantation are likely to be driven by rising purchasing power and the attendant demand for health care of higher quality by the sector of the population who can afford it. Achieving equity, transparency and ethical practice in the provision of organ transplantation – especially in a setting of low economic and health system development and high out-of-pocket expenditures – will require the implementation of appropriate regulatory frameworks and oversight.

## Policy implications

Our analysis of the global diffusion of transplantation capacity indicates that, in general, transplanting and non-transplanting Member States are currently differentiated on the basis of physician-to-population ratios and health expenditure per capita – but not by gross national income per capita. Although affluent countries are the earliest adopters of new medical technologies, the availability of such technologies gradually becomes less dependent on economic factors over time.^8^ Rising incomes, the spread of health insurance, lifestyle factors adding to the burden of illness and ageing populations have increased demand for the treatment of end-stage organ failure in low- and middle-income countries. In addition, actors in the public and private health sectors may have an interest in increasing the supply of transplant services in low- and middle-income countries, and linkages to facilitate skills transfer across the international medical community have been actively contributing to the development of local transplantation capacities. For these and other reasons, the practice of organ transplantation has now diffused across all income strata. Therefore, it is appropriate for ministries of health in all jurisdictions – including low- and middle-income countries – to develop policies with respect to organ donation and transplantation. An immediate requirement is a legal framework to protect donors and recipients and to regulate medical practice. The next step is the development of specialist surgeons, physicians and nurses.

The transition from a transplantation programme that only involves living donor transplantation to one that includes deceased donor transplantation remains linked with income per capita. Deceased donor transplantation can only proceed where there is a legal framework in place for the declaration of death and the lawful removal of organs from deceased persons for the purpose of transplantation. The elements of a comprehensive national transplantation programme include: (i) a legal framework and regulatory oversight, (ii) an adequately resourced deceased donor programme, (iii) a waiting list of candidates who are allocated organs irrespective of gender, ethnicity or social status, (iv) an ethical living donor programme; and (v) clinical practices consistent with international standards.^29^ For countries seeking to increase rates of deceased donor transplantation, the key reforms of the Spanish Model – i.e. centralized coordination, orientation of organ recovery around transplant coordinators and systematization of donor identification and organ recovery – have been effective in a diverse range of countries (Box 2). For small countries, the development of organ donation and transplantation capacity may necessitate regional cooperation.

Box 2 **Policy implications for the development of solid organ donation and transplantation**

- Even where transplantation capacity does not currently exist, epidemiological, demographic and economic transitions are increasing the demand for organ transplantation. Therefore, prospective health policies addressing the role of living and deceased donor transplantation in the health system, beginning with appropriate legislative frameworks, are warranted for all countries.
- For those countries seeking to improve rates of deceased donor transplantation, the efficacy of the Spanish Model of organ donation and transplantation has now been demonstrated across a diverse range of countries. The model’s key reforms include centralization of the coordination of organ donation and transplantation under an official authority, reorientation of organ recovery around transplant coordinators and the systematization of donor identification and organ recovery.
- Low- and lower-middle-income countries are capable of providing living donor transplantation programmes, given a willingness to allocate resources and personnel to this goal and the existence of one or more highly trained individuals. The transition from a transplant programme based only on living donors to one that also includes deceased donor transplantation requires a substantially greater investment of resources. It is also dependent on engagement with policy-makers to remove legal impediments to the recovery of organs from dead donors, and engagement with the public to increase public acceptance of deceased donation.
- For health systems that are underdeveloped and for countries where out-of-pocket and private payments for health-care services are high, there is a particular need for health policies that uphold the principles of equity and transparency in the provision of transplantation, and for legislation prohibiting unethical practices.
- Governments are accountable for the implementation of transplantation programmes in which the opportunity to benefit is shared equitably across the population. Achieving this requires: (i) appropriate legislation, regulation and oversight, (ii) registries to monitor activities and outcomes and to ensure transparency of practices; and (iii) the optimization of activities – consistent with competing demands on health resources – by focusing on specialist training, particularly the training of transplant coordinators and the implementation of a structured professional network that incorporates continuous training and performance assessment.

Finally, in presenting overall regional trends, we have not commented on intraregional variation in transplantation activities or on the spatial, socioeconomic, racial and gender disparities in access to transplantation that exist within individual Member States. As the diffusion of the practice of transplantation continues, equity of access will be a major challenge. Catch-up growth, market integration, increased personal income and savings and epidemiological and demographic transitions – all of which have combined to increase the burden of organ failure in developing countries while simultaneously increasing the wealth of upper-income households – have the potential both to increase demand for transplantation and to exacerbate inequities in access within low- and middle-income countries.^30^ With the integration of organ donation and transplantation into national health systems, governments are accountable for establishing programmes in which the opportunity to benefit from transplantation is shared equally across the population.^29^ Legislation, regulatory oversight and the monitoring and transparent reporting of organ donation and transplantation practices through national registries are key to this accountability.
